# Supplementary figures and images for: Cre/lox regulated conditional rescue and inactivation with zebrafish UFlip alleles generated by CRISPR-Cas9 targeted integration
Source: eLife. 2022 Jun 17;11:e71478. doi: 10.7554/eLife.71478 (PMC9270027; doi:10.7554/eLife.71478)

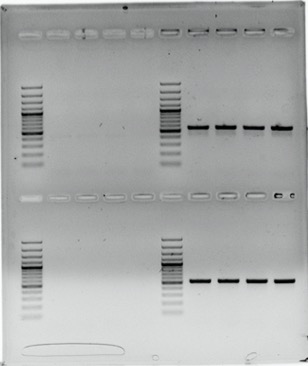

Supplement: Source data 1. [file elife-71478-data1.zip › Liu et al gel image files Source Data/Figure 8 - figure supplement 1 gel .jpg]

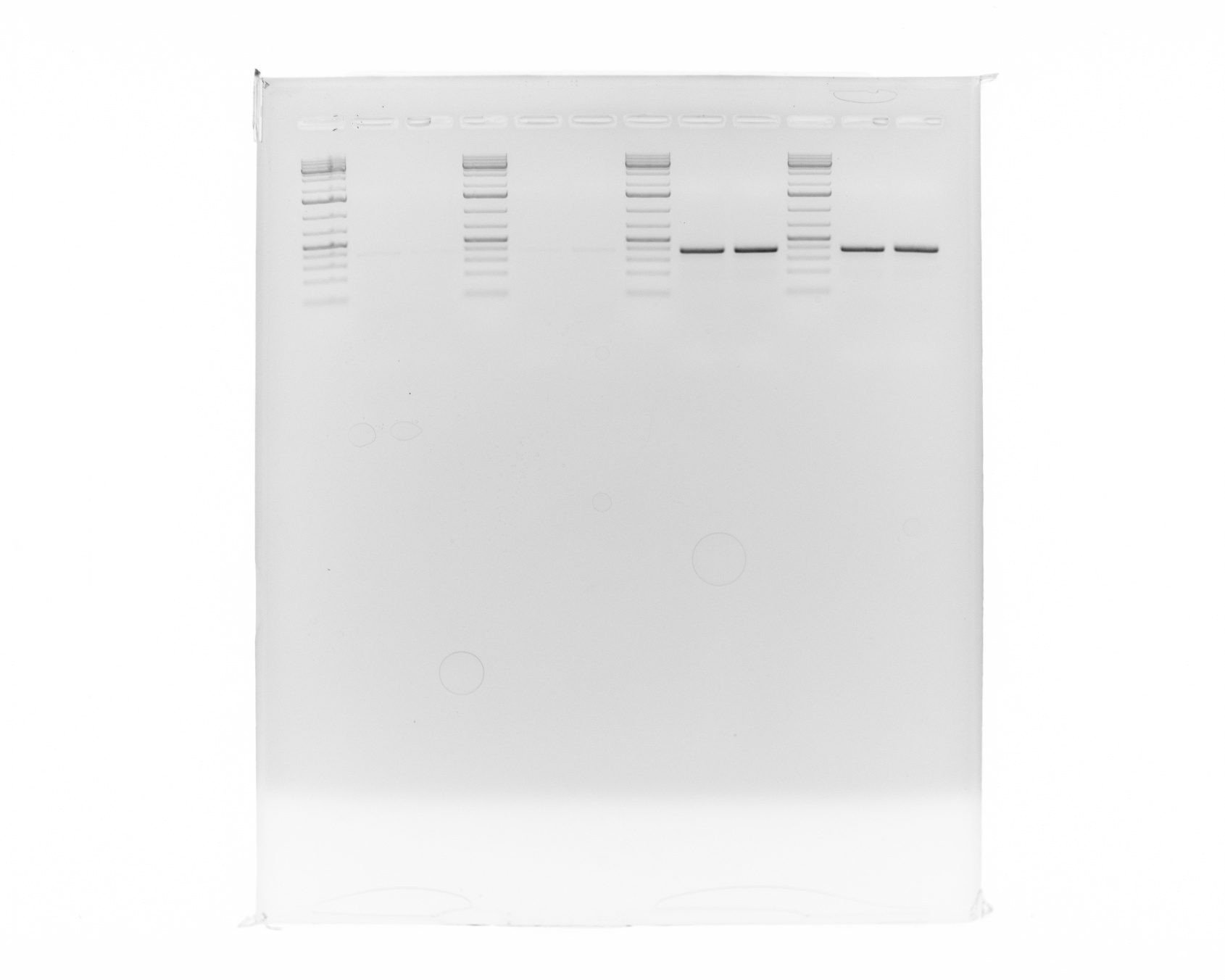

Supplement: Source data 1. [file elife-71478-data1.zip › Liu et al gel image files Source Data/Figure 6 - Figure supplement 1N.jpg]

Figure 2 rb1 intron 6 gRNA test gel

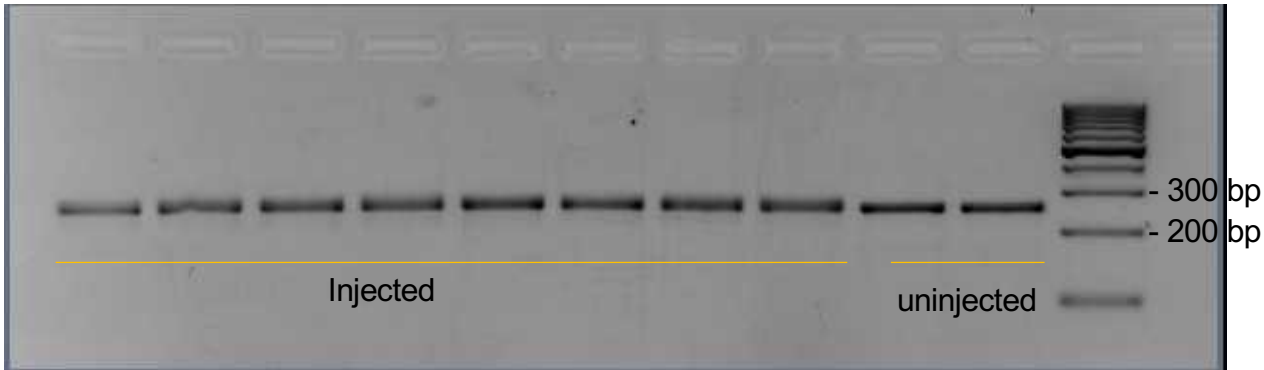

Supplement: Source data 1. [file elife-71478-data1.zip › Liu et al gel image files Source Data/Figure 2 rb1 intron 6 gRNA test gel.pdf]

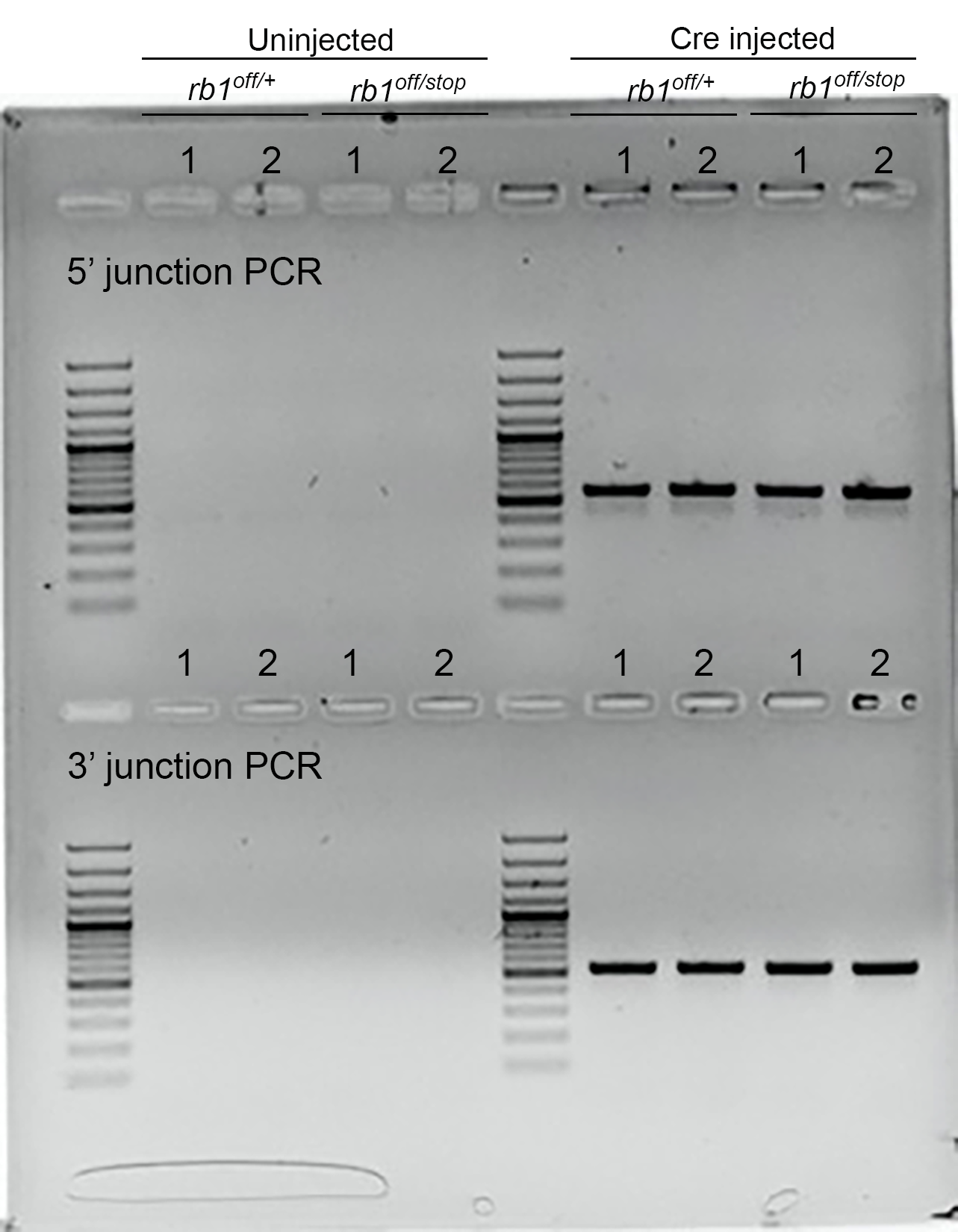

Supplement: Source data 1. [file elife-71478-data1.zip › Liu et al gel image files Source Data/Figure 8 - figure supplement 1 gel annotated.tif]

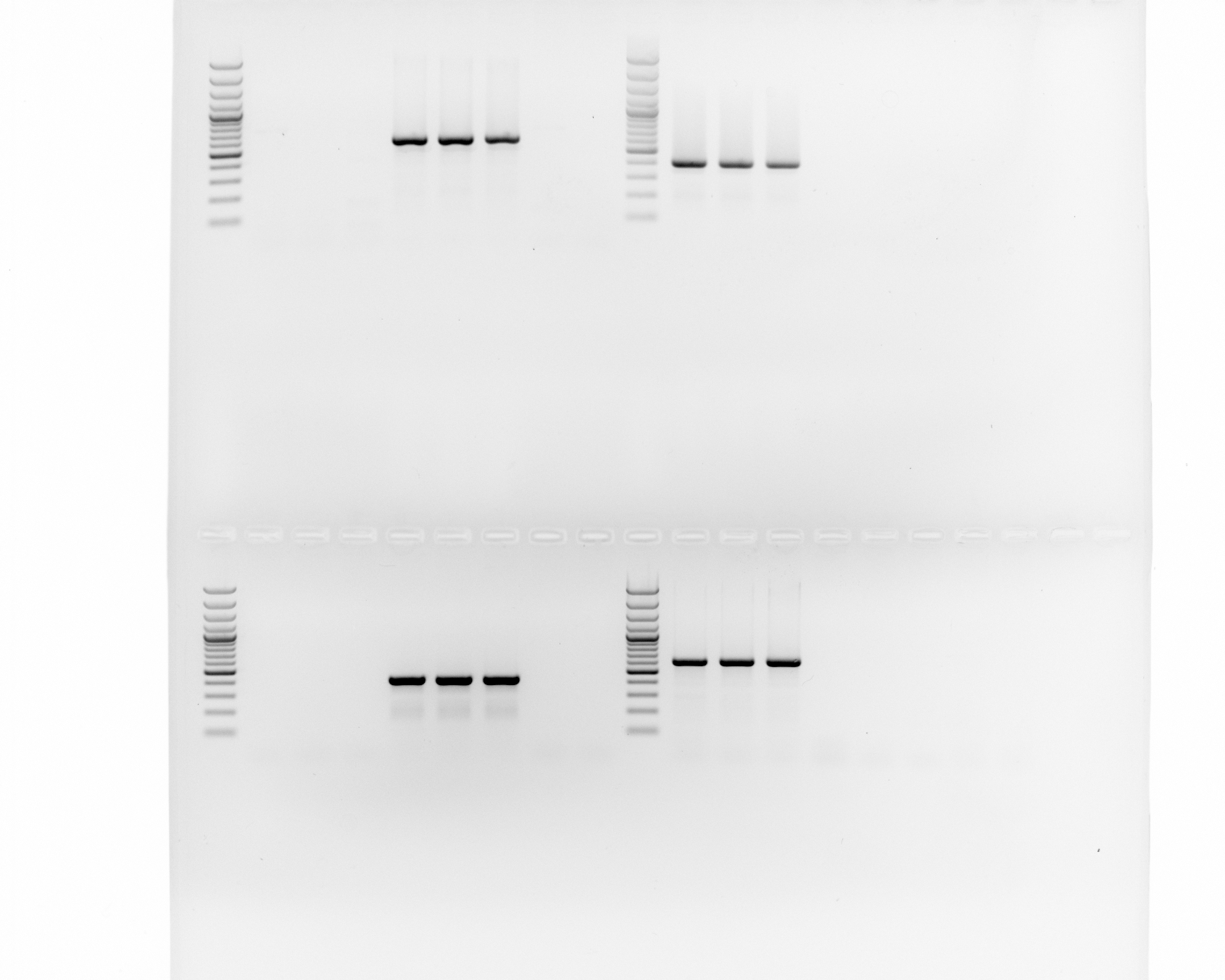

Supplement: Source data 1. [file elife-71478-data1.zip › Liu et al gel image files Source Data/Figure 3 gel.tif]

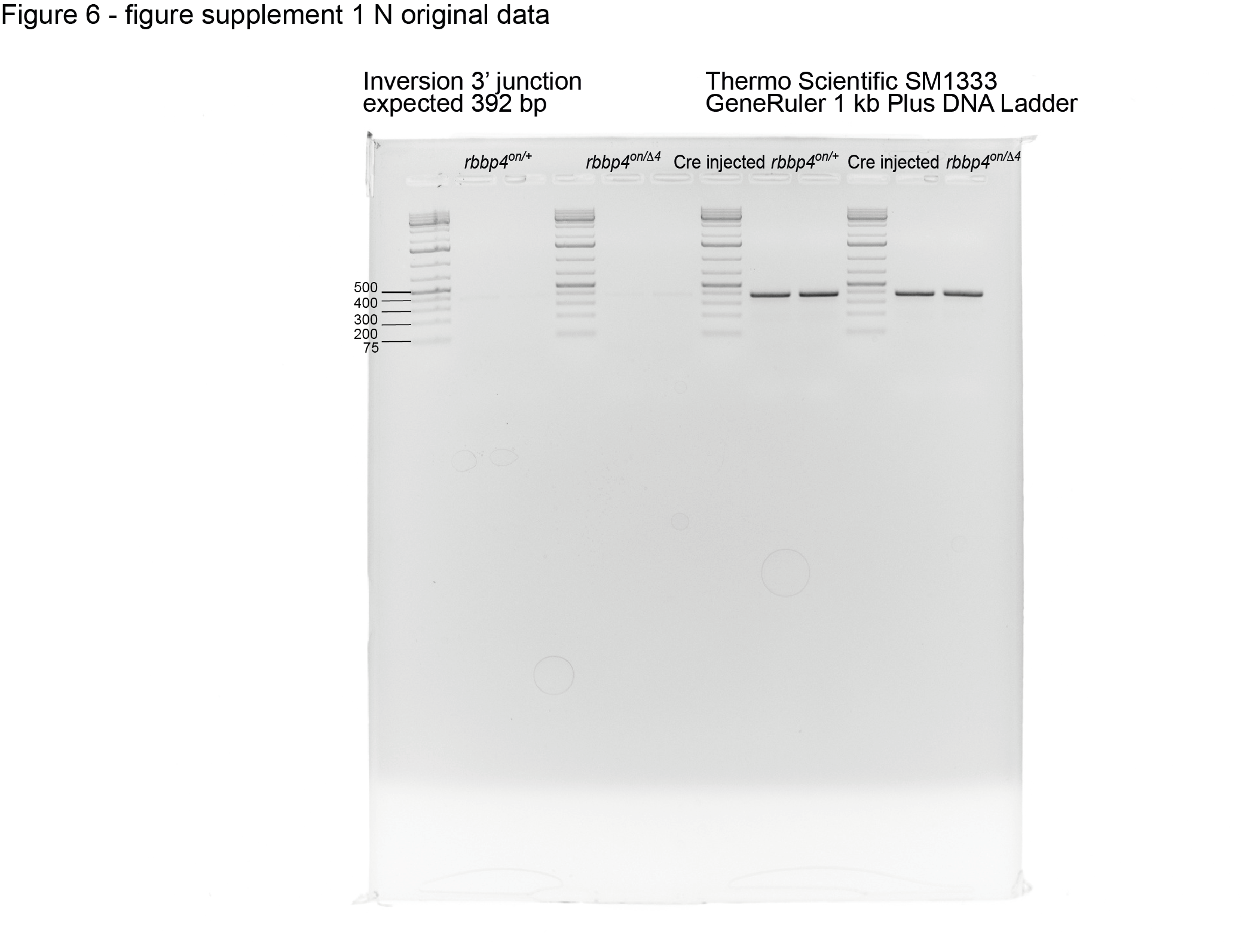

Supplement: Source data 1. [file elife-71478-data1.zip › Liu et al gel image files Source Data/Figure 6 - Figure supplement 1N.png]

Figure 9 – figure supplement 1 gels

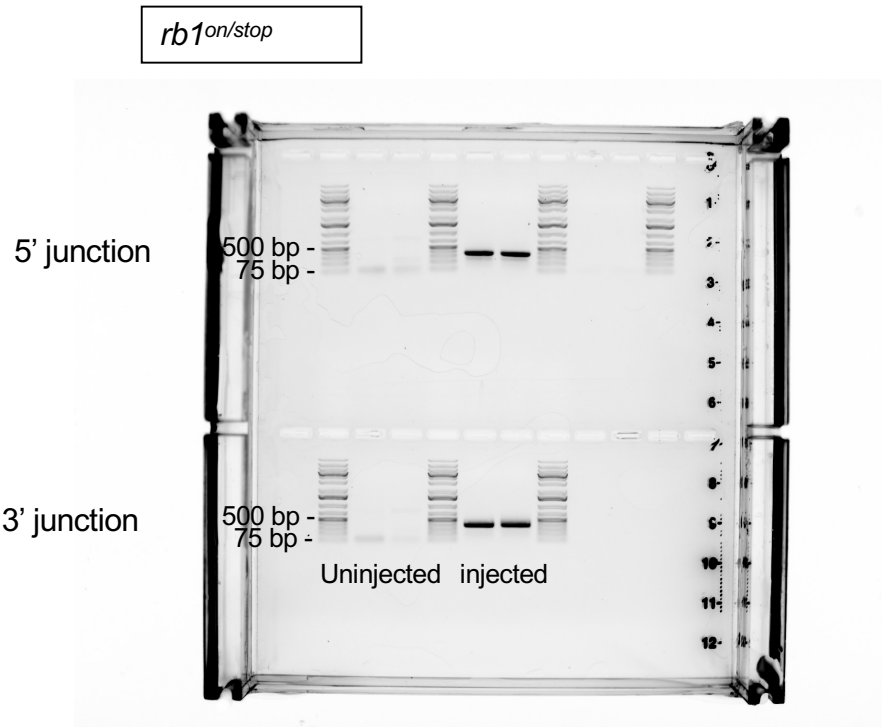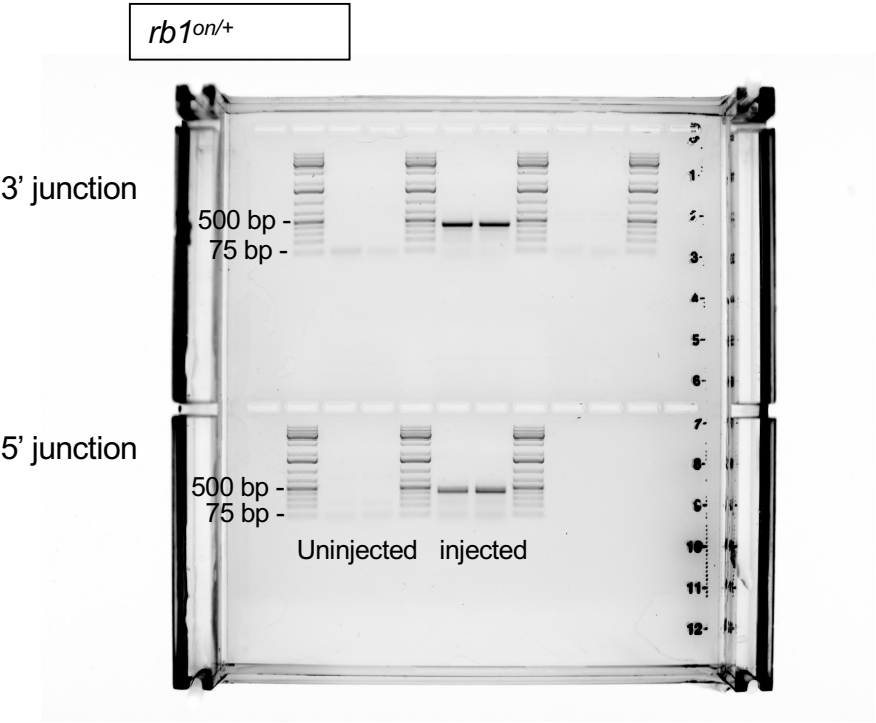

Supplement: Source data 1. [file elife-71478-data1.zip › Liu et al gel image files Source Data/Figure 9 - figure supplement 1 gels.pdf]

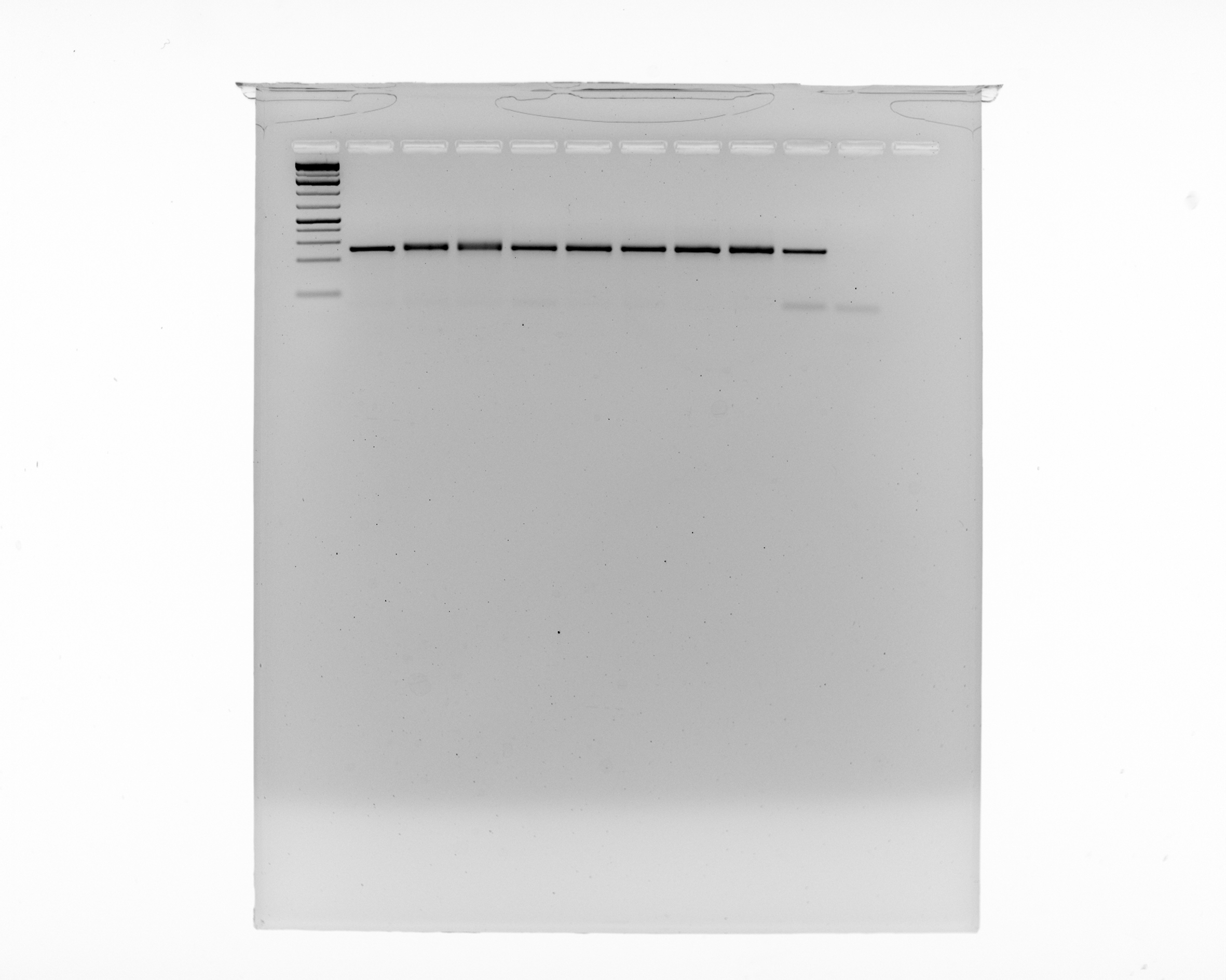

Supplement: Source data 1. [file elife-71478-data1.zip › Liu et al gel image files Source Data/Figure 2 rbbp4 intron 4 gRNA test gel.tif]

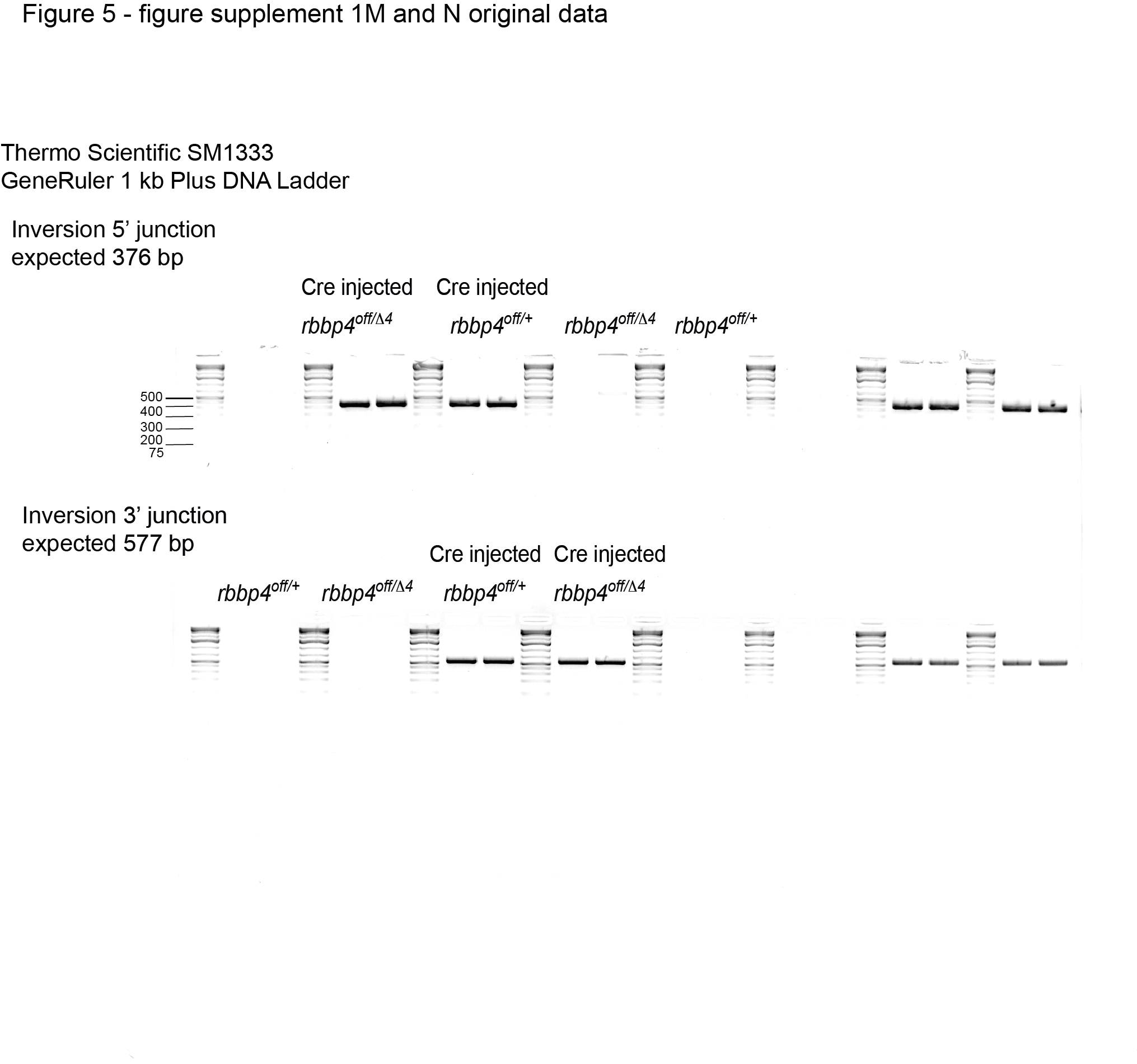

Supplement: Source data 1. [file elife-71478-data1.zip › Liu et al gel image files Source Data/Figure 5 - Figure supplement 1M and 1N.png]

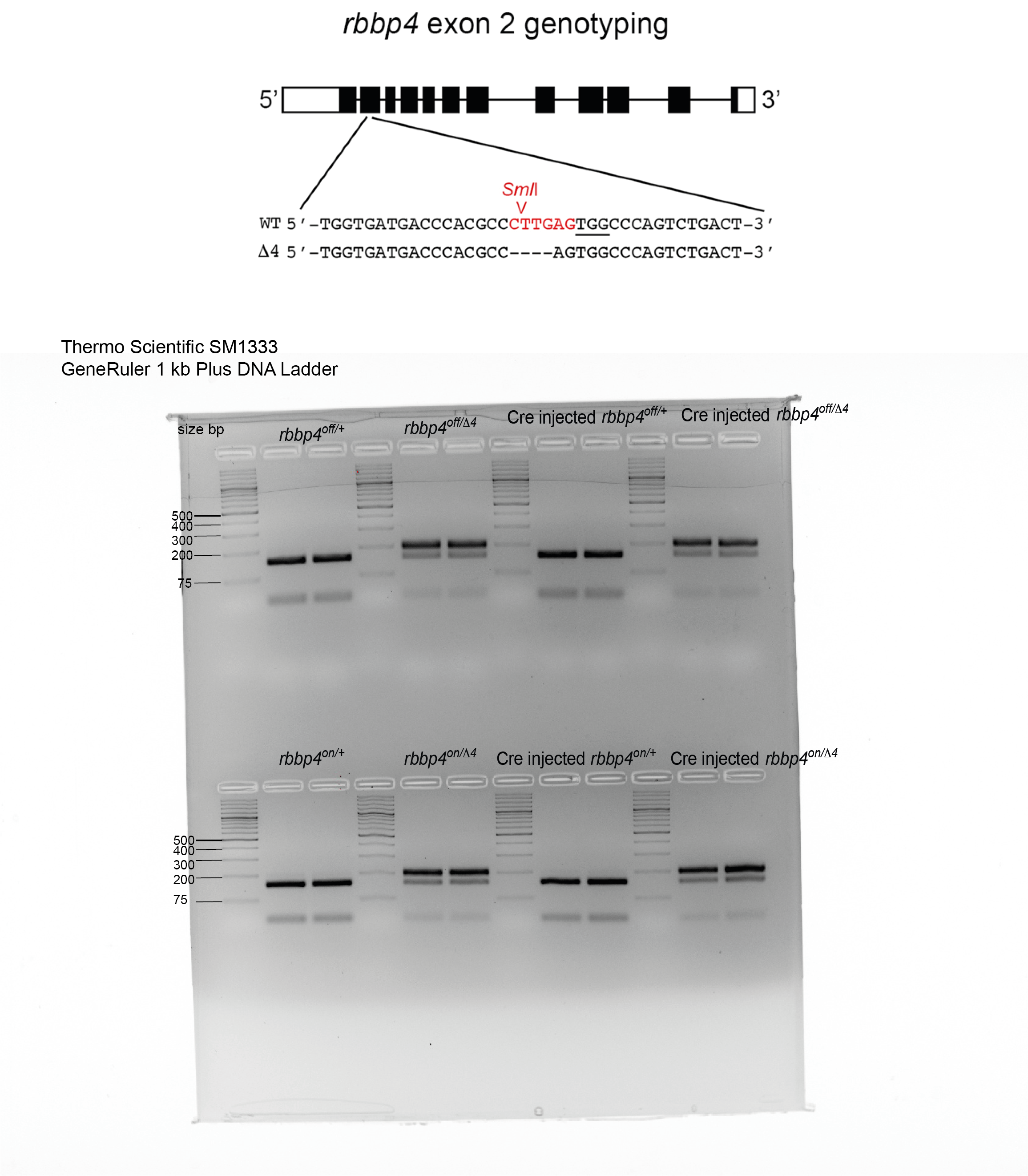

Supplement: Source data 1. [file elife-71478-data1.zip › Liu et al gel image files Source Data/Figure 5 and 6 - Figure supplement 1 O rbbp4 exon2 genotyping.png]

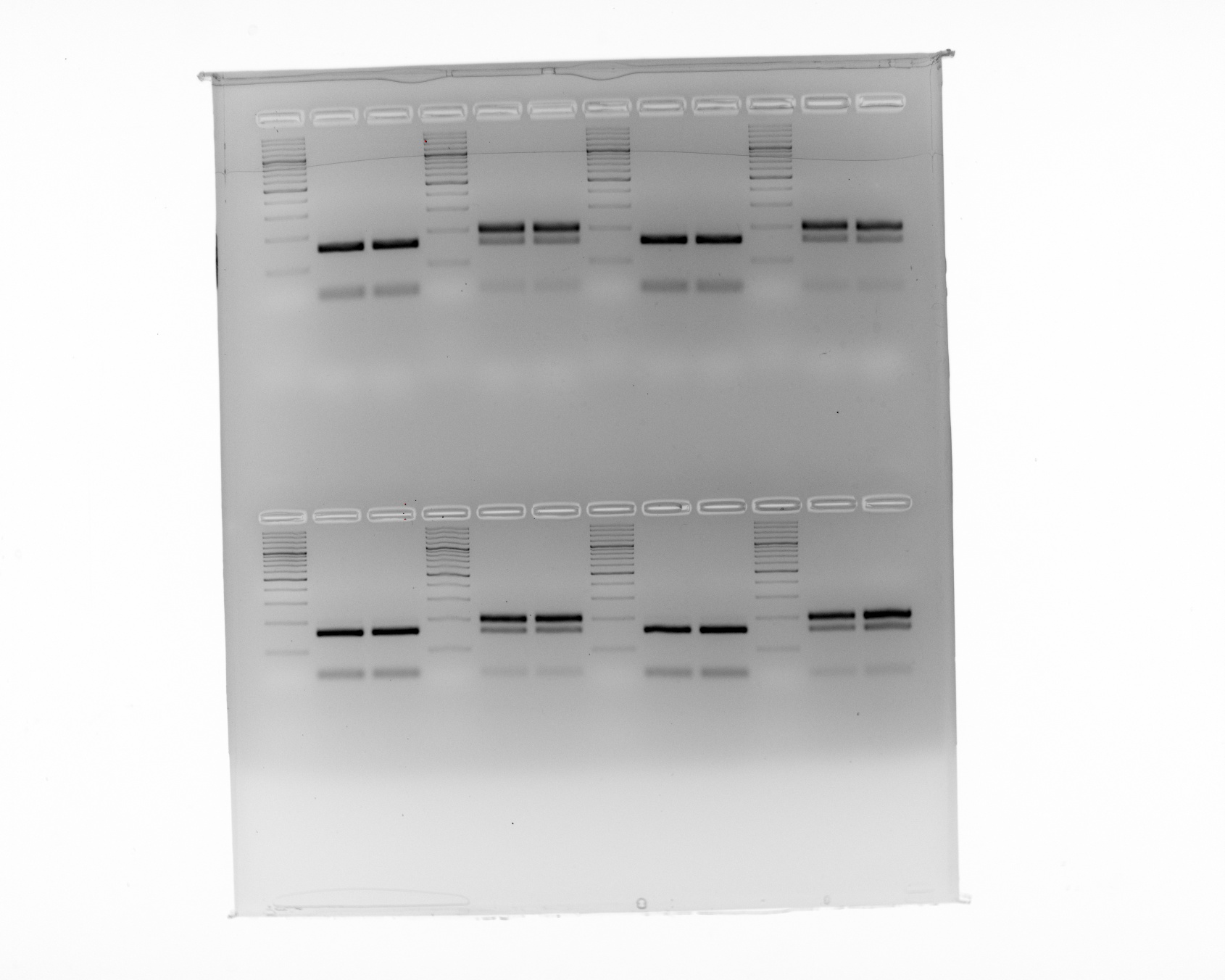

Supplement: Source data 1. [file elife-71478-data1.zip › Liu et al gel image files Source Data/Figure 5 and 6 - Figure supplement 1 O rbbp4 exon2 genotyping.jpg]

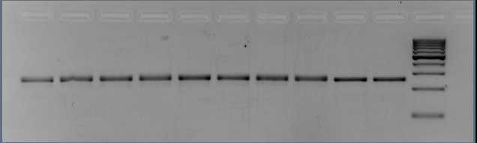

Supplement: Source data 1. [file elife-71478-data1.zip › Liu et al gel image files Source Data/Figure 2 rb1 intron 6 gRNA gel test.png]

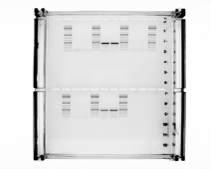

Supplement: Source data 1. [file elife-71478-data1.zip › Liu et al gel image files Source Data/Figure 9 - figure supplement gel 2.png]

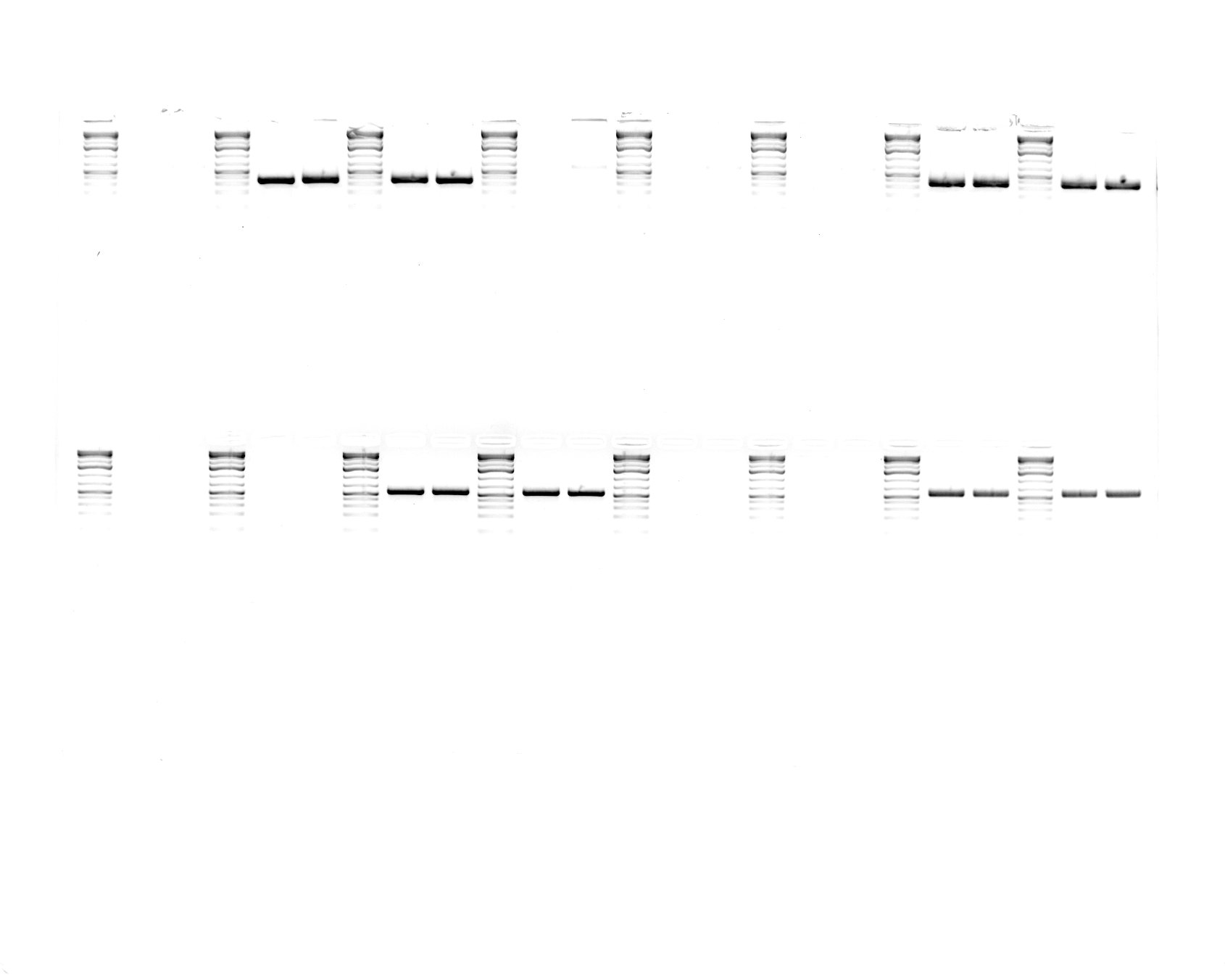

Supplement: Source data 1. [file elife-71478-data1.zip › Liu et al gel image files Source Data/Figure 5 Figure supplement 1M and 1N.jpg]

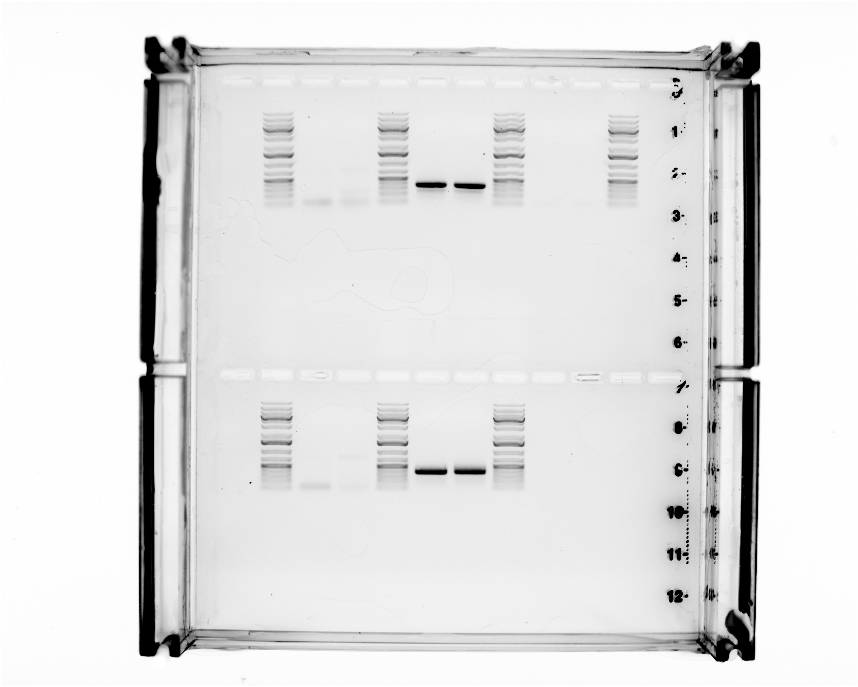

Supplement: Source data 1. [file elife-71478-data1.zip › Liu et al gel image files Source Data/Figure 9 - figure supplement gel 1.png]

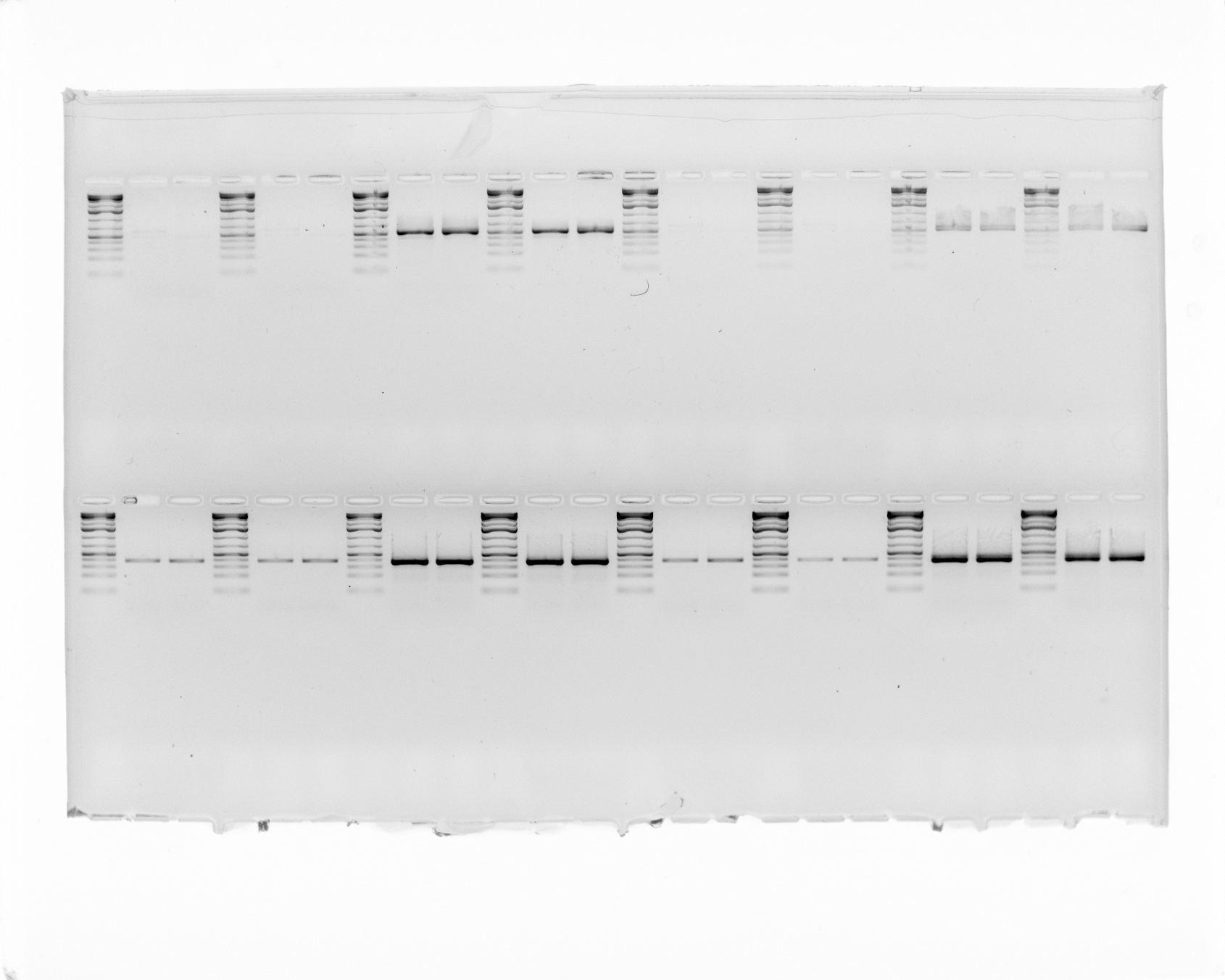

Supplement: Source data 1. [file elife-71478-data1.zip › Liu et al gel image files Source Data/Figure 6 - Figure supplement 1M.jpg]

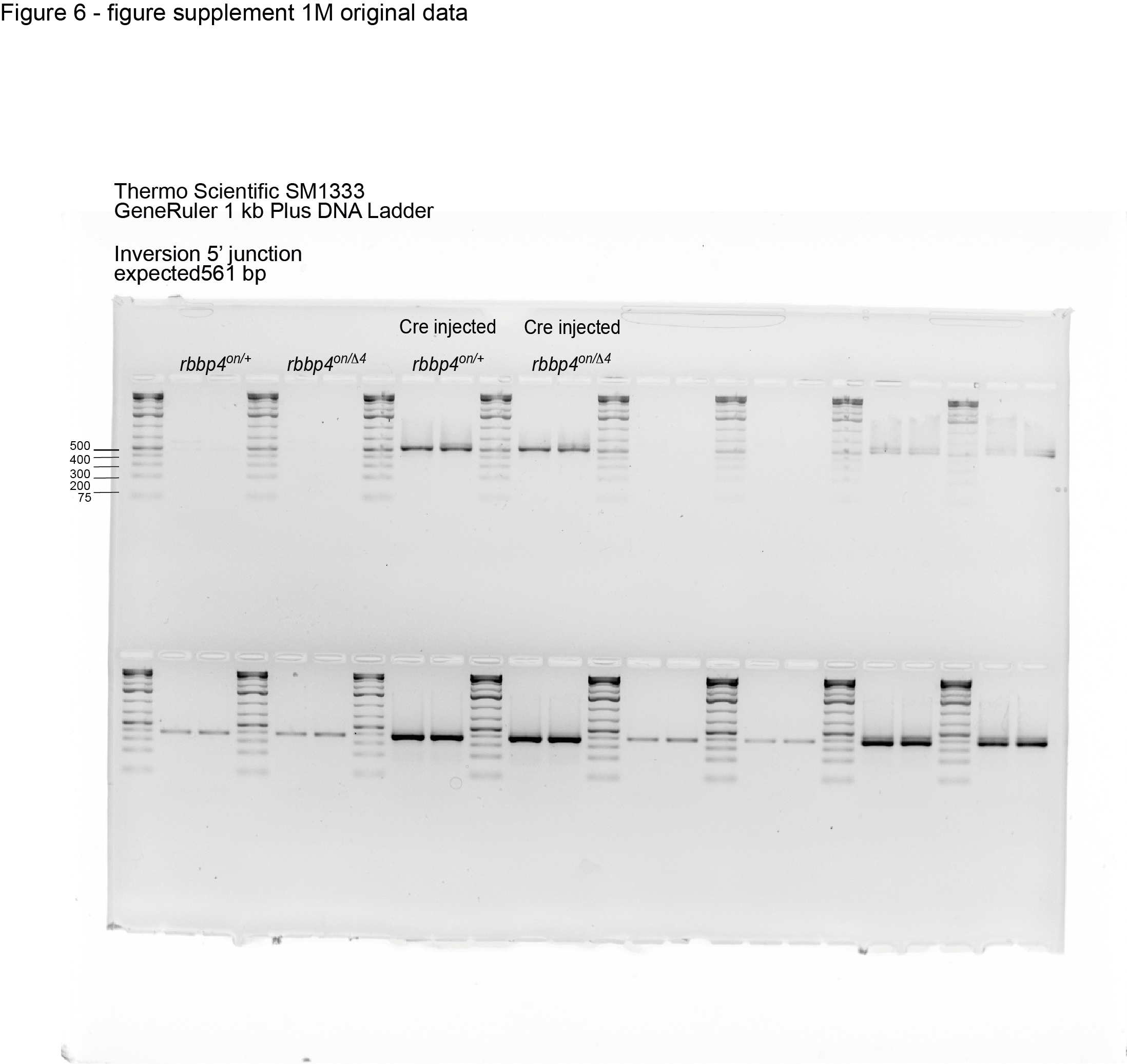

Supplement: Source data 1. [file elife-71478-data1.zip › Liu et al gel image files Source Data/Figure 6 - Figure supplement 1M.png]

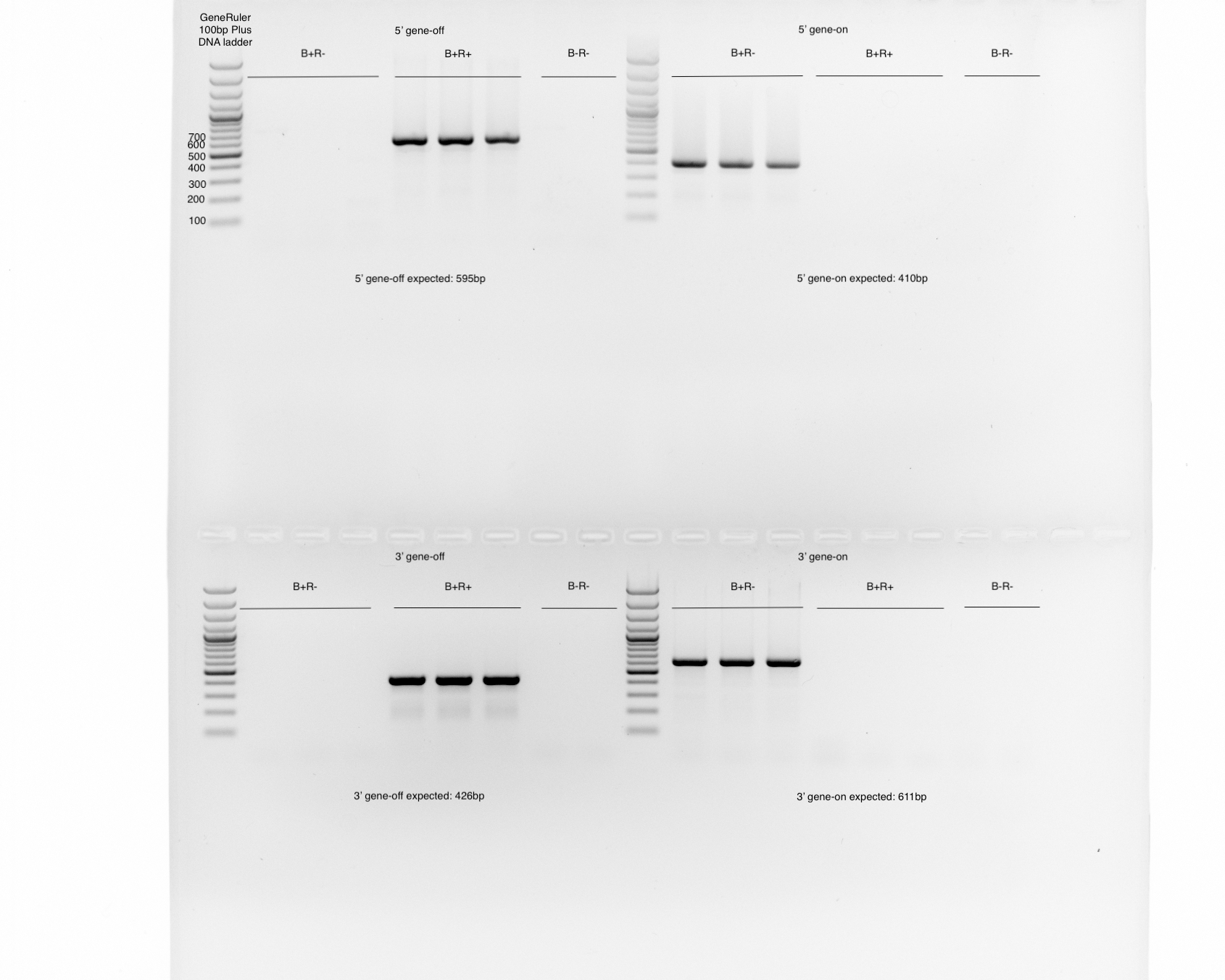

Supplement: Source data 1. [file elife-71478-data1.zip › Liu et al gel image files Source Data/Figure 3 gel annotated.tif]
