# Supplementary material for: Cre/lox regulated conditional rescue and inactivation with zebrafish UFlip alleles generated by CRISPR-Cas9 targeted integration
Source: eLife. 2022 Jun 17;11:e71478. doi: 10.7554/eLife.71478 (PMC9270027; doi:10.7554/eLife.71478)
Supplement: Transparent reporting form [file elife-71478-transrepform1.docx]

***eLife’s* transparent reporting form**

We encourage authors to provide detailed information *within their submission* to facilitate the interpretation and replication of experiments. Authors can upload supporting documentation to indicate the use of appropriate reporting guidelines for health-related research (see [EQUATOR Network](http://www.equator-network.org/%20)), life science research (see the [BioSharing Information Resource](https://biosharing.org/)), or the [ARRIVE guidelines](http://www.plosbiology.org/article/info:doi/10.1371/journal.pbio.1000412) for reporting work involving animal research. Where applicable, authors should refer to any relevant reporting standards documents in this form.

If you have any questions, please consult our Journal Policies and/or contact us: [editorial@elifesciences.org](mailto:editorial@elifesciences.org).

**Sample-size estimation**

- You should state whether an appropriate sample size was computed when the study was being designed
- You should state the statistical method of sample size computation and any required assumptions
- If no explicit power analysis was used, you should describe how you decided what sample (replicate) size (number) to use

Please outline where this information can be found within the submission (e.g., sections or figure legends), or explain why this information doesn’t apply to your submission:

The sample size was not computed when the study was designed. Approximately 100 embryos were injected per targeting experiment, with the goal of raising 30 founders to adulthood to screen for germline transmission. Two factors can impact founder fish sample size. First, the number of embryos injected in a given morning, which can be small due to low fertilization rates. Second, fluctuation in the survival rate of juvenile fish to adulthood due to paramecia culture variability impacts the number of founders available to screen for germline transmission. Genotypic, phenotypic and quantification analyses were performed in triplicate (three biological replicates of each confirmed genotype) which provided sufficient statistical power. Source data files for all quantitative measurements have been provided

**Replicates**

- You should report how often each experiment was performed
- You should include a definition of biological versus technical replication
- The data obtained should be provided and sufficient information should be provided to indicate the number of independent biological and/or technical replicates
- If you encountered any outliers, you should describe how these were handled
- Criteria for exclusion/inclusion of data should be clearly stated
- High-throughput sequence data should be uploaded before submission, with a private link for reviewers provided (these are available from both GEO and ArrayExpress)

Please outline where this information can be found within the submission (e.g., sections or figure legends), or explain why this information doesn’t apply to your submission:

Each injection experiment was repeated at least three times. For injection experiments, replicates were both biological (different batches of embryos and needles) and technical (different injections in the same batch of embryos). Biological and technical replicates for quantitative PCR are described in the materials and methods. Conditional gene inactivation, phenotypic analysis, and quantification were performed on three biological replicates as described in the figure legends and materials and methods. Figure legends describe the statistical methods used and n=sample size. Source data files for all quantitative measurements presented in each figure have been provided in the section “Additional Data Files”.

**Statistical reporting**

- Statistical analysis methods should be described and justified
- Raw data should be presented in figures whenever informative to do so (typically when N per group is less than 10)
- For each experiment, you should identify the statistical tests used, exact values of N, definitions of center, methods of multiple test correction, and dispersion and precision measures (e.g., mean, median, SD, SEM, confidence intervals; and, for the major substantive results, a measure of effect size (e.g., Pearson's r, Cohen's d)
- Report exact p-values wherever possible alongside the summary statistics and 95% confidence intervals. These should be reported for all key questions and not only when the p-value is less than 0.05.

Please outline where this information can be found within the submission (e.g., sections or figure legends), or explain why this information doesn’t apply to your submission:

N value, statistical test, and mean +- SEM were indicated in each figure legend. P values were reported in the figure legends.

(For large datasets, or papers with a very large number of statistical tests, you may upload a single table file with tests, Ns, etc., with reference to sections in the manuscript.)

**Group allocation**

- Indicate how samples were allocated into experimental groups (in the case of clinical studies, please specify allocation to treatment method); if randomization was used, please also state if restricted randomization was applied
- Indicate if masking was used during group allocation, data collection and/or data analysis

Please outline where this information can be found within the submission (e.g., sections or figure legends), or explain why this information doesn’t apply to your submission:

For conditional gene inactivation experiments, samples were first sorted based on expression of fluorescence reporters and gross morphological phenotype, followed by molecular analysis to confirm genotype. For quantitative RT-PCR, 25 random embryos were selected, individually genotyped, then 5 individual head samples of the same genotype pooled for RNA extraction. No masking was used in group allocation.

**Additional data files (“source data”)**

- We encourage you to upload relevant additional data files, such as numerical data that are represented as a graph in a figure, or as a summary table
- Where provided, these should be in the most useful format, and they can be uploaded as “Source data” files linked to a main figure or table
- Include model definition files including the full list of parameters used
- Include code used for data analysis (e.g., R, MatLab)
- Avoid stating that data files are “available upon request”

Please indicate the figures or tables for which source data files have been provided:

Additional data were provided in the supplemental tables
